# Saline Treatment of Dysentery

**Published:** 1853-09

**Authors:** 


*Saline Treatment of Dysentery.*

SEVERAL cases, which have come under our observation recently, of successful treatment of Dysentery with chloride of sodium, sulphate of magnesia, and other salts in small doses, have convinced us that these remedies are not used as often as they might be with benefit in a class of cases in which portal congestion is the immediate cause of too frequent and free discharges of bloody and serous fluid from the intestines.

In a former nunber of this journal, we endeavoured to show that alkalies and their compounds are the proper remedies in all cases of torpidity of the liver, resulting from a deficiency of alkalies as compared with fatty acids for the formation of bile.

Portal congestion from torpidity of the liver, caused by deficiency in the alkaline constituents of the blood, is doubtless one of the most frequent causes of dysentery and serous diarrhœa; hence it is that the substances under consideration are in many cases the most efficient and prompt remedies.

The cases which have been treated successfully by these remedies under our observation are not numerous, but sufficient in number to justify us in calling the attention of our readers to the subject.

One of the prescriptions that have been found most efficient in dysentery is—Chloride of Sodium ʒss, Sulph. Morph. gr.⅓ in powder or dissolved in mucilege of gum arabic, repeated every four or six hours. H.

